# Immunomodulatory Potential of Diuretics

**DOI:** 10.3390/biology10121315

**Published:** 2021-12-11

**Authors:** Paweł Bryniarski, Katarzyna Nazimek, Janusz Marcinkiewicz

**Affiliations:** Department of Immunology, Jagiellonian University Medical College, 18 Czysta Street, 31-121 Krakow, Poland; katarzyna.nazimek@uj.edu.pl (K.N.); janusz.marcinkiewicz@uj.edu.pl (J.M.)

**Keywords:** diuretics, immunomodulatory effect, antihypertensive drugs, immunology, humoral response, cellular response

## Abstract

**Simple Summary:**

In the elderly population, it is important to find the right anti-inflammatory and pro-inflammatory balance to avoid the impairment of the immune system, which becomes weaker with age. This age-dependent weakness of the immune system can increase the risk of serious infections and premature death. This review describes diuretics (substances that promote urination) and their effect on the immune system. The effect on the immune system of this group of drugs was noted in patients suffering from hypertension, observed under experimental conditions with laboratory animals. The cause of hypertension is strongly connected to chronic inflammation. Most diuretics are anti-inflammatory, but depending on the drug, the effects may vary. This topic is highly significant in medical practice regarding the treatment of patients with associated diseases with chronic inflammatory etiology, including arterial hypertension or chronic heart failure. In obese patients and patients with allergies, the anti-inflammatory effect is beneficial because the immune system is too strongly activated. In this article, we try to provide doctors practical advice regarding the type of diuretic that should be used in patients with specific clinical problems and additional diseases.

**Abstract:**

In this review, diuretics and their immunomodulatory functions are described. The effects on the immune response of this group of drugs are reported in patients suffering from hypertension and under experimental conditions involving animal models and cell line studies. The pathogenesis of hypertension is strongly connected to chronic inflammation. The vast majority of diuretics modulate the immune response, changing it in favor of the anti-inflammatory response, but depending on the drug, these effects may differ. This topic is significantly important in medical practice regarding the treatment of patients who have coexisting diseases with chronic inflammatory pathogenesis, including hypertension or chronic heart failure. In patients with metabolic syndrome, allergies, or autoimmune disorders, the anti-inflammatory effect is favorable, because of the overstimulation of their immune system. Otherwise, in the geriatric population, it is important to find the proper anti- and pro-inflammatory balance to avoid an enhancement of immune response suppression, which can result in reducing the risk of serious infections that can occur due to the age-diminished function of the immune system. This article is intended to facilitate the selection of an antihypertensive drug that depends on the patient’s immune situation.

## 1. Background

Hypertension is one of the most common diseases in adults [1,2]. Treatment of hypertension is a major challenge for the population, because approximately half of hypertensive patients do not adequately control their blood pressure. 

There are two definitions of arterial hypertension: European and American. According to the definition of the American College of Cardiology/American Heart Association (ACC/AHA), grade one hypertension can be diagnosed when the systolic blood pressure is at least 130 mmHg and/or the diastolic blood pressure is at least 80 mmHg [3].

The definition of The European Society of Cardiology/European Society of Hypertension (ESC/ESH) is more liberal. According to this definition, we can diagnose grade one hypertension if the systolic blood pressure is at least 140 mmHg and the diastolic blood pressure is at least 90 mmHg [4,5].

There are two types of hypertension: primary and secondary. The exact etiology of essential (primary) hypertension is unclear. However, there are several risk factors that are strongly associated with essential hypertension development, such as older age, obesity [6,7], family history (hypertension occurs twice as often in patients when at least one of their parents had hypertension) [8,9], race (hypertension occurs more often in African and African American patients) [10,11], reduced nephron number, high-sodium diet [12,13,14], excessive alcohol consumption [15], and physical inactivity [7,16]. On the contrary, the major causes of secondary hypertension are medications (high doses of estrogen [17], nonsteroidal anti-inflammatory agents (NSAIDs) [18,19,20,21], antidepressants, corticosteroids, tyrosine kinase inhibitors, etc.), drug use (e.g., methamphetamines and cocaine), primary renal disease [22], primary aldosteronism [23,24,25,26], renovascular hypertension [27,28], obstructive sleep apnea [29,30,31,32,33], pheochromocytoma [34,35,36], Cushing’s syndrome [37,38], other endocrine disorders (hypothyroidism [39], hypertyroidism [40], and hyperparathyroidism [41,42,43,44]), as well as coarctation of the aorta [45].

Hypertensive patients are very commonly medicated with diuretics, which differ in their therapeutic potency. Mild diuretics include osmotic agents (mannitol), carbonic anhydrase inhibitors (acetazolamide), and potassium-sparing diuretics (spironolactone, amiloride). Meanwhile, moderate-potency diuretics include thiazides and thiazide-like agents, and strong diuretics include loop diuretics.

The substances that promote urination also differ in terms of the site of knitting in the nephron [46]. Carbonic anhydrase inhibitors and osmotic diuretics act in the proximal tubule [47,48], while potassium-sparing diuretics target the distal nephron (including the connecting tubule and collecting duct). Thiazide and thiazide-like diuretics act in the distal tubule [49,50,51,52,53], while loop diuretics, as the name suggests, affect the loop of Henle [54,55,56]. Since hypertension is an inflammation-related disease, it is important to unravel the putative immunomodulatory properties of the medications used for treating affected patients. This review summarizes the current knowledge on the immunomodulatory properties of diuretic drugs.

## 2. Results and Discussion

### 2.1. Loop Diuretics

Two of most popular loop diuretics, furosemide and torasemide, have similar mechanisms of action, but the latter seems to cause lesser side effects. Furosemide inhibits the reabsorption of chloride ions in the nephron, in the ascending part of the Henle loop. This, in turn, inhibits the reabsorption of sodium ions, which are excreted, pulling water with it. In addition, this also increases the excretion of magnesium, calcium, and potassium, but decreases the excretion of uric acid. Furosemide is used to treat acute left ventricular failure, pulmonary edema, and exacerbations of chronic heart failure. The diuretic effect is used in the treatment of nephrotic syndrome, acute and chronic renal failure (with severe reduced Glomerular Filtration Rate (GFR) when thiazide drugs cease to be effective), liver cirrhosis, in cases of resistance to thiazide hypertension, intoxication during forced diuresis, hypercalcemia, severe circulatory failure, and prevention of cerebral edema.

The strong effect of loop diuretics may result in large electrolyte disturbances, such as hypovolemia, hyponatremia, hypomagnesemia, hypokalemia, hypocalcaemia, and chlorine deficiency alkalosis. Moreover, an orthostatic drop in blood pressure, including collapse, may occur. Loop diuretics require (due to their high potency) appropriate indications and strict electrolyte control, especially when using digitalis glycosides.

Thus far, furosemide has been found to reduce the levels of tumor necrosis factor alpha (TNF-α; in a dose-dependent manner) [57], IL-6, IL-8 [58], and IL-10 [59], but does not show any significant effect on IL-1β/IL-2 [57] (Table 1). Similarly, furosemide reduces the intracellular concentrations of interleukin (IL)-6 and TNF-α [60]. This drug does not affect the effectiveness of phagocytosis and the number of phagocytic neutrophils [61], but seems to partially prevent the activation of pulmonary macrophages and bronchial epithelial cells by reducing the production of superoxide anions [62]. Inhaled furosemide has been demonstrated to decrease inflammatory cytokines and airway hyper-reactivity in asthmatic subjects [63], but its influence on allergic asthmatic reactions in mice is ambiguous. Depending on the experimental settings, furosemide has been shown to either increase the infiltration of T lymphocytes [64], or to reduce the infiltration of pulmonary inflammatory cells [65]. Furosemide and spironolactone reduce the migration of leukocytes through endothelial cell monolayers [66]. Furosemide increases the expression of the genes responsible for the pro-inflammatory response: acute phase proteins (hemeoxygenase-1 and metallothionein-1), stress proteins (C/Ebp homologous protein and growth arrest and DNA damage-induced protein), inflammatory cytokines (IL-1β), chemokines (macrophage inflammatory protein 2 and murine keratinocyte-derived chemokine), and apoptosis (early growth factor response and BCL2 related protein X) in mice [67]. Furosemide administered with kanamycin and lipopolysaccharide enhances the inflammatory response to hair cell damage and results in increased hearing loss [68]. In patients with nasal polyposis, treatment with furosemide does not significantly affect the number of inflammatory cells, but significantly reduces swelling in previously untreated patients [69]. Furosemide reduces the production of prostaglandins in cultured human epithelial cells from nasal polyps in vitro, making intranasal furosemide a candidate for the treatment of edema formation in nasal polyps [70,71]. Cardio-renal syndrome is characterized as a dysfunction of mutually influencing systems, such as excretory and cardiovascular, and is based on hemodynamic changes, neurohormonal activation, inflammation, oxidative stress, iron disorders, anemia, and disturbances in the mineral metabolism. One of the treatments for this syndrome is furosemide, which modifies the underlying factors [72]. In a study on rats, it was proved that in ischemic reperfusion, furosemide combined with sitagliptin can significantly inhibit the elevation of TNF-α, NF-κB, caspase-3, NO, and iNOS, and can increase the activity of glutathione and antioxidant enzymes in the kidney and heart tissues [73]. In patients who suffer from heart failure, furosemide acts as anti-inflammatory by reducing TNF-α, IL-1-β, IL-6, and the natriuretic peptides ANP and BNP [74,75]. Similar observations (i.e., decrease in the IL-6 and IL-8 concentrations) after treatment with furosemide have been observed in a septic newborn, due to *Staphylococcus epidermidis* infection [76], without affecting the expression of the IL-6 and IL-8 genes [77]. Interestingly, furosemide used in the intoxication of a toxic fungus (*Cortinarius speciosissimus*) in a rat research model had no effect on the parameters of inflammation [78]. In the rat model, it was shown that ethacrynic acid and dichlorothiazide reduced increases in skin vascular permeability, and that long-term therapy with furosemide, dichlorothiazide, and ethacrynic acid reduces the dry weight of the inflammatory granuloma, which demonstrates anti-inflammatory properties [79].

After SARS-CoV-2 infection, the inflammatory response of the host often results in excessive secretion of inflammatory cytokines (IL-6 and TNF-α especially), which transforms into a detrimental “cytokine storm”. In this clinical situation, furosemide inhibits IL-6 and TNF-α, and may be an agent ameliorating the cytokine storm of COVID-19 [80]. Similarly, torasemide seems to possess anti-inflammatory properties, as shown in the experimental autoimmune myocarditis. This long-acting loop diuretic reduces the progression of myocarditis to dilated cardiomyopathy [81]. Torasemide decreases the levels of the beta1 transforming protein in rats, improves myocardial function parameters, and also inhibits left ventricle (LV) fibrosis [82]. Torasemide reduces the level of the oxidative stress marker p67phox and reduces the expression of the mineralocorticoid receptor [83].

Bumetanide is a potent loop diuretic that acts as an inhibitor of sodium–potassium–chloride cotransporter 2 (NKCC2) and its NKCC1 isoform. This drug is indicated for the treatment of high blood pressure, pulmonary edema, edema associated with renal and heart failure, nephrotic syndrome, and cirrhosis. Bumetanide may cause cramps, hypotension, headache, nausea, and hepatic encephalopathy in patients with concomitant liver disease. Bumetanide decreases the activation of inflammatory cells. This drug, which is administered as an intra-tracheal spray, also reduces tissue inflammation and acute lung injury [84]. 

Ethacrynic acid is an ototoxic drug with loop diuretic activity. It causes hearing damage that may be irreversible. Unlike other loop diuretics, it does not have a sulfonamide structure and can be used in patients who are hypersensitive (allergic) to sulfonamides. The indications are the same as for other loop diuretics. On the one hand, etacrynic acid improves macrophage functioning under hyperoxic conditions [85], while on the other hand, it inhibits the NF-kappaB signaling pathway in lipopolysaccharide-activated macrophages [86], suggesting that this drug may be used in the future for the treatment of diseases associated with excessive inflammation. In addition, the use of ethacrynic acid reduces the expression of pro-inflammatory cytokine IL-6 and iNOS expression in the intestinal wall, and decreases gastrointestinal stasis in postoperative mouse intestinal obstruction [87].

**Table 1:** The effect of loop diuretics on selected parts of the immune system. Abbreviations: TNF-α, tumor necrosis factor alpha; IL, interleukin; NF-κB, nuclear factor kappa-light-chain-enhancer of activated B cells; NO, nitric oxide; iNOS, inducible nitric oxide synthase; C/Ebp, CCAAT/enhancer binding proteins; BCL2, B-cell lymphoma 2; p67phox, neutrophil cytosolic factor 2.

Drug	Immunological Mechanism (Reference)
Furosemide	Reduction in: - Levels of TNF-α [57,74,75,80]; - Levels of interleukines IL-6 [74,75,76,80], IL-8 [58,76], and IL-10 [59]; - Intracellular concentrations of IL-6 and TNF-α [60]; - Production of superoxide anions and, as a result, prevention of the activation of pulmonary macrophages and bronchial epithelial cells [62]; - Levels of inflammatory cytokines and airway hyper-reactivity in asthmatic subjects (taken as an inhaler) [63]; - Migration of leukocytes through endothelial cell monolayers [66]; - Swelling in patients with nasal polyposis (previously untreated) [69]; - Production of prostaglandins in cultured human epithelial cells from nasal polyps in vitro, making the intranasal form of this drug a candidate for the treatment of edema formation in nasal polyps [70,71]; - Oxidative stress in cardio-renal syndrome [72]; - Inhibition if the elevation of TNF-α, NF-κB, caspase-3, NO, and iNOS, increasing the activity of glutathione and antioxidant enzymes in kidney and heart tissue (used in rats) [73]; - Dry weight of the inflammatory granuloma, which demonstrates anti-inflammatory properties—taken in long-term therapy with furosemide, dichlorothiazide, and ethacrynic acid in a rat model [79]. No significant effect on: - Levels of IL-1β/IL-2 [57], or reduces the IL-1β concentration [74,75]; - Effectiveness of phagocytosis and the number of phagocytic neutrophils [61]; - Allergic asthmatic reactions in mice (ambiguous: depending on the experimental settings, it has been shown to either increase the infiltration of T lymphocytes [64], or to reduce the infiltration of pulmonary inflammatory cells [65]); - Number of inflammatory cells in patients with nasal polyposis [69]; - Expression of the IL-6 and IL-8 genes [77]; - Parameters of inflammation (used in the intoxication of a toxic fungus in a rat research model) [78]. Increase in: - Expression of genes responsible for the pro-inflammatory response: acute phase proteins (hemeoxygenase-1 and metallothionein-1), stress proteins (C/Ebp homologous protein and growth arrest and DNA damage-induced protein), inflammatory cytokines (IL-1β), chemokines (macrophage inflammatory protein 2 and murine keratinocyte derived chemokine), and apoptosis (early growth factor response and BCL2 related protein X) in mice [67]; - Inflammatory response to hair cell damage and increased hearing loss (administered with kanamycin and lipopolysaccharide) [68].
Torasemide	Reduction in: - Progression of myocarditis to dilated cardiomyopathy (seems to possess anti-inflammatory properties) [81]; - Levels of the beta1 transforming protein in rats [82]; - Levels of the oxidative stress marker p67phox and expression of the mineralocorticoid receptor [83].
Bumetanide	Reduction in: - Activation of inflammatory cells [84].
Ethacrynic acid	Reduction in: - Dry weight of the inflammatory granuloma, which demonstrates anti-inflammatory properties—taken in long-term therapy with furosemide, dichlorothiazide, and ethacrynic acid in a rat model [79]; - NF-kappaB signaling pathway in lipopolysaccharide-activated macrophages [86], while improving macrophage functioning under hyperoxic conditions [85]; - Expression of pro-inflammatory cytokine IL-6 and iNOS in the intestinal wall and gastrointestinal stasis in postoperative mouse intestinal obstruction [87].

### 2.2. Potassium-Sparing Diuretics

The most commonly used potassium-sparing diuretic is spironolactone, which, analogously to eplerenone, increases the amount of excreted urine, sodium ions, and chloride, clearly reducing the amount of excreted potassium and hydrogen ions. Spironolactone indications are primary and secondary hyperaldosteronism, cardiac oedema, hepatic and renal origin, idiopathic edema, advanced myocardial insufficiency (NYHA grade IV), nephrotic syndrome (in the case of unsatisfactory effects of treatment of the primary disease), premenstrual syndrome, adjuvant treatment of myasthenia gravis, and hirsutism. The most common side effects of spironolactone are hyperkalemia, nipple sensitivity to touch, gynecomastia, erectile dysfunction, hypertrophy and breast tenderness in women, menstrual disorders, headaches, and drowsiness.

Spironolactone exerts beneficial effects in patients with inflammatory disorders, such as congestive heart failure and chronic arthritis, because it inhibits the production of TNF-α, IL-6, IL-8, nitric oxide (NO), prostaglandin E2, monocyte chemoattractant protein-1 (MCP-1), granulocyte macrophage colony-stimulating factor, and IFN-gamma, without affecting the release of IL-1 β [88,89,90,91,92] (Table 2). Spironolactone, as an antagonist of the mineralocorticoid receptor, prevents acute lung injury and fibrosis by inhibiting the M2 polarization of alveolar macrophages [93]. Through its antagonism to aldosterone, this drug induces an anti-inflammatory effect and blocks the polarization of CD4+ Th17 cells, but does not alter the B cell population and most of T lymphocyte subpopulations, apart from increasing the number of naive helper T cells [94,95,96].

Spironolactone, by reducing the release of monocyte chemoattractant (MCP) protein and transforming growth factor (TGF)-β1, as well as macrophage and CD4+ T cell infiltration, reduces inflammation and peritoneal fibrosis [97,98] Moreover, a diet rich in salt causes the increased expression of M1 macrophage markers (iNOS and IFN-γ), without affecting the expression of M2 macrophage markers (IL-10, ArgI, and ED2 protein content). Spironolactone used in these patients reduces the negative impact of this diet in terms of stimulating the macrophage population [98]. Spironolactone, in renal transplant patients, was shown not to significantly affect the markers of endothelial dysfunction nor those of inflammation, except in a subgroup analysis of diabetic patients, where spironolactone decreased nitrite when compared to the placebo [99]. Treatment of hypertension with spironolactone by sodium removal reduces endothelial glycocalyx dysfunction, inflammation, NETosis, and coagulation disorders, leading to the improvement in vascular health and the diastolic function of the heart [100]. Interestingly, spironolactone (in combination with bromhexine) has successfully been used in the new coronavirus (SARS-CoV-2) infection by achieving faster normalization of the clinical condition, a one and a half times faster temperature reduction, and a shorter hospitalization time, which allows us to speculate about its anti-inflammatory properties [101]. In dogs suffering from heart failure assisted by ventricular pacing, spironolactone prevented the overexpression of the inflammatory cytokine gene (IL-6 and TNF-α) [102]. In a randomized, controlled trial in patients with pulmonary arterial hypertension, spironolactone was shown to improve endothelial function and reduce inflammation [103]. Similar observations were also made in a mouse model, where treatment with spironolactone decreased the ability to produce superoxides in the cerebral arteries, as well as the mRNA expression of the pro-inflammatory cytokines CCL7, CCL8, and IL-1β in the brain [104]. Spironolactone and eplerenone attenuate bleomycin-induced pneumonia and fibrosis in rodent models, and attenuate the increase in neutrophils and bronchoalveolar lavage macrophages (BALs) [105]. However, in double-blind, randomized, placebo-controlled studies, it has been shown that spironolactone does not change the markers of inflammation or endothelial dysfunction, but only decreases NT-proBNP [106]. Spironolactone in mice significantly improves inflammation relief and accelerates wound healing upon exposure to nitrogen mustard (an alkylating agent that causes severe skin damage). Spironolactone inhibits the expression of iNOS in the skin and decreases the expression of matrix metallopeptidase 9, CCL-2, IL-1α and IL-1β, and the number of local pro-inflammatory M1 macrophages, resulting in an increase in the M2/M1 ratio in the wound microenvironment [107]. A previous meta-analysis proved that spironolactone reduces the markers of fibrosis and inflammation, including NIIINP, PICP, hs-CRP, and TNF-α [108]. In the TOPCAT Biorepository Study (Treatment of Preserved Cardiac Function Heart Failure with an Aldosterone Antagonist Trial), for patients with symptomatic heart failure (HF), ejection fraction (EF) ≥ 45%, and elevated natriuretic peptide levels or prior hospitalization for HF, spironolactone did not affect CRP levels [109]. In patients who suffered from acute kidney injury, administration of spironolactone protected against conversion to chronic kidney disease and had an anti-inflammatory effect [110].

Spironolactone and eplerenone (which is a more selective mineralocorticoid receptor antagonist) reduce the vasculitis and cardiovascular risk in hypertensive and diabetic patients [111], as well as inflammation, fibrosis, and oxidative stress in the kidneys [112]. In vitro studies have shown that spironolactone inhibits fibrogenesis in TGF-β-stimulated human colon myofibroblasts, but spironolactone therapy significantly increases the mortality in rodents with inflammatory bowel fibrosis, suggesting that spironolactone may be harmful during enteritis [113].

Eplerenone is an aldosterone receptor blocker that is used to treat heart failure. It is recommended as a first-line treatment in patients with left ventricular systolic dysfunction (EF < 40%) and clinical signs of heart failure after a heart attack, in co-therapy with beta-blockers [114]. Eplerenone transiently increases the concentration of monocytic proteins such as 1-chemoattractant, IL-1β, IL-10, and IL-4, which improves macrophage functioning, and therefore has a beneficial effect on the heart after infarction, as it increases neovascularization of the infarcted area [115].

Eplerenone inhibits inflammation (macrophage and monocyte infiltration) and fibrosis (by reducing the level of IL-1β), reduces oxidative stress, and promotes alternative activation in macrophages [116,117,118,119]. Eplerenone has a beneficial effect in patients with metabolic syndrome, as it prevents excessive weight gain and fat storage and improves glucose intolerance and insulin resistance. This effect is achieved by blocking the mineralocorticoid receptor on macrophages. Eplerenone reduces the levels of TNF-α, IL-6 and TGF-β, ROS, MMP-2, and IL-1β [120,121,122]. Eplerenone, which is used in diabetic nephropathy in mice, reduces the markers of inflammation and oxidative stress, as well as the expression of TNF-α, MCP-1, Nox2, and p47phox, as well as the levels of renal thiobarbituric acid reactive substances (TBARSs) [123]. In rats, in the myocardium after myocardial infarction, spironolactone and eplerenone lower the levels of Gal-3 (Galectin-3, which plays an important role in cell-cell adhesion and macrophage activation), TGF-β [124], and IL-1β [125], and also significantly decreases CD80-positive pro-inflammatory M1 macrophages, as well as increases CD163-positive anti-inflammatory M2 macrophages in infarction. Interestingly, the use of one of the angiotensin receptor antagonists also reduces the apoptosis of myocytes in the peri-infarction zone by 40–50% [126]. Studies in human embryonic kidney cells (HEK 293) have shown that spironolactone has an anti-inflammatory effect that is independent of aldosterone, unlike eplerenone, of which the basis of its anti-inflammatory effect is anti-aldosterone activity [127]. In the HIV-infected human population, eplerenone is used as an anti-inflammatory by reducing the levels of IL-6 and hs-CRP [128]. In mice, eplerenone reduces T cell accumulation and IFN-γ production [129]. Spironolactone and eplerenone block ICAM-1 and CTGF transcription by inhibiting SGK1 and NF-κB, which allows the inhibition of mesangial fibrosis and glomerulonephritis [130]. Eplerenone in mice with viral myocarditis reduces the presence of monocytes/macrophages, oxidative stress, and the risk of cardiac fibrosis [131]. Spironolactone and eplerenone in rats reduce the concentration of reactive oxygen intermediates (ROIs) and CRP, which are increased by aldosterone [132]. In septic patients, spironolactone improves survival and alleviates kidney damage by inhibiting inflammation and apoptosis [133]. Spironolactone reduces the secretion of inflammatory mediators (IL-6, monocyte chemoattractant protein-1, IL-18, IL-27, and IFN-γ) and plasminogen activator inhibitor (PAI)-1 in human aortic endothelial cells [134]. At the cellular level, aldosterone receptor antagonists block oxidative stress signaling pathways, leading to an increase in bioavailable nitric oxide, a reduction in inflammation, the inhibition of cell proliferation, and a decrease in the rate of fibrosis [135]. In the treatment of tendinopathy, spironolactone inhibits the IL-1β-induced overexpression of inflammatory factors [136]. Spironolactone reduces organ damage caused by a high-salt diet by blocking T helper 17 activation and downregulating regulatory T cells [137].

Amiloride is a diuretic that increases the excretion of sodium ions and water from the body, but diminishes the excessive excretion of potassium. Thus far, amiloride has been shown to reduce the production of IL-1β, IL-6, IL-8, IL-12, and TNF-α. The anti-inflammatory effect of the drug is used in the treatment of the inflammatory component of shock, RSV infection (used in inhalation), or in the protection of the heart against reperfusion damage [138,139,140,141].

**Table 2:** The effect of potassium-sparing diuretics on selected parts of the immune system. Abbreviations: NO, nitric oxide; PGE2, prostaglandin E2; MCP-1, monocyte chemoattractant protein-1; GM-CSF, granulocyte macrophage colony-stimulating factor; TGF, transforming growth factor; MMP 9, matrix metallopeptidase 9; CCL-2, C-C ligand 2; NIIINP, aminoterminal propeptide of type III procollagen; PICP, procollagen type I carboxyterminal propeptide; hs-CRP, high-sensitivity CR; Gal-3, galectin-3, ROIs, reactive oxygen intermediates; CRP, C-reactive protein; PAI-1,plasminogen activator inhibitor 1; IL, interleukin; IFN-γ, interferon gamma; iNOS, inducible nitric oxide synthase; HF, heart failure; CD, cluster of differentiation; TBARSs, thiobarbituric acid reactive substances.

Drug	Immunological Mechanism (Reference)
Spironolactone	Reduction in: - Migration of leukocytes through endothelial cell monolayers [66]; - Production of TNF-α, IL-6, IL-8, NO, PGE2, MCP-1, GM-CSF, and IFN-γ (so exerts beneficial effects in patients with inflammatory disorders, such as congestive heart failure and chronic arthritis) [88,89,90,91,92]; - M2 polarization of alveolar macrophages, so prevents acute lung injury and fibrosis [93]. - Polarization of CD4+ Th17 cells [94,95,96]; - Release of MCP and TGF-β1, as well as macrophage and CD4+ T cell infiltration, inflammation [100], and peritoneal fibrosis [97,98]; - Expression of M1 macrophage markers (iNOS and IFN-γ) [98]; - Level of nitrite in renal transplant patients with diabetes [99]; - Overexpression of the inflammatory cytokine gene (IL-6 and TNF-α) [102]; - Inflammation in patients with pulmonary arterial hypertension (improves endothelial functioning) [103]; - Production of superoxides in the cerebral arteries, as well as the mRNA expression of the pro-inflammatory cytokines CCL7, CCL8, and IL-1β in the brain [104]; - Increase in neutrophils and bronchoalveolar lavage macrophages in rats with pneumonia [105]; - Expression of iNOS (inhibition) in the skin and expression of MMP-9, CCL-2, IL-1α, and IL-1β (decrease) and the number of local pro-inflammatory M1 macrophages (decrease), resulting in an increase in the M2/M1 ratio in the wound microenvironment [107]; - Markers of fibrosis and inflammation, including NIIINP, PICP, hs-CRP, and TNF-α [108]; - Inflammation in patients suffering from acute kidney injury [110]; - Inflammation and apoptosis in septic patients and, as a result, improved survival and alleviation of kidney damage [133]; - Secretion of inflammatory mediators (IL-6, MCP-1, IL-18, IL-27, and IFN-γ) and PAI-1 in human aortic endothelial cells [134]; - Overexpression of inflammatory factors (inhibits IL-1β induction) [136]; - Organ damage caused by a high-salt diet by blocking T helper 17 activation and downregulation of regulatory T cells [137]. Non-significant effect on: - Release of IL-1β [88,89,90,91,92]; - B cell population and most T lymphocyte subpopulations [94,95,96]; - Expression of M2 macrophage markers (IL-10, ArgI, and ED2 protein content) [98]; - Markers of endothelial dysfunction or inflammation in renal transplant patients [99]; - Markers of inflammation and endothelial dysfunction [106]; - CRP levels in patients with symptomatic HF [109]. Increase in: - Number of naive helper T cells [94,95,96].
Spironolactone and Eplerenone	Reduction in: - Inflammation, fibrosis, and oxidative stress in the kidneys [112]; - Levels of Gal-3, TGF-β, IL-1β, and CD80-positive pro-inflammatory M1 macrophages [124,125]; - Apoptosis of myocytes in the peri-infarction zone by 40%–50% [126]; - Mesangial fibrosis and glomerulonephritis [130]; - Concentration of ROIs and CRP [132]; - Oxidative stress signaling pathways, leading to an increase in bioavailable nitric oxide, inflammation, cell proliferation, and the rate of fibrosis [135]. Increase in: - CD163-positive anti-inflammatory M2 macrophages in infarction [124,125].
Eplerenone	Reduction in: - Inflammation (macrophage and monocyte infiltration), fibrosis (by reducing the level of IL-1β), and oxidative stress [116,117,118,119]; - Levels of TNF-α, IL-6 and TGF-β, ROS, MMP-2, and IL-1β [120,121,122]; - Markers of inflammation and oxidative stress, as well as the expression of TNF-α, MCP-1, Nox2, and p47phox, and the levels of renal TBARSs in diabetic nephropathy in mice [123]; - Levels of IL-6 and hs-CRP [128]; - T cell accumulation and IFN-γ production [129]; - Presence of monocytes/macrophages, oxidative stress, and the risk of cardiac fibrosis in viral myocarditis [131]. Increase in: - Concentration of monocytic proteins 1-chemoattractant, IL-1β, IL-10, and IL-4, which improve macrophage functioning [115]; - Promotion of alternative activation in macrophages [116,117,118,119].
Amiloride	Reduction in: - Production of IL-1β, IL-6, IL-8, IL-12, and TNF-α [138,139,140,141].

### 2.3. Carbonic Anhydrase Inhibitors

Carbonic anhydrase inhibitors are weak diuretics, with low efficiencies—around 4%. These are short-acting drugs, due to the tolerance phenomenon and the ability to compensate for the supply of hydrogen ions without carbonic anhydrase. Increased diuresis arises as a result of the inability to exchange sodium–hydrogen ions, due to the lack of the latter, which results in an increased loss of sodium, potassium, and water. There are four carbonic anhydrase inhibitors: acetazolamide, methazolamide, brinzolamide, and dorzolamide. Indications for use are glaucoma, edema in heart failure or which is drug-induced, acute altitude sickness (the drug shortens acclimatization time), post-traumatic brain edema, paroxysmal dizziness, premenstrual syndrome, and epilepsy (petit mal in children, grand mal, and mixed forms). These drugs may also express anti-inflammatory properties, since certain studies have shown that acetazolamide reduces TNF-α production by mouse macrophages, and thus may suppress inflammatory reactions [142,143]. Acetazolamide used in acute mountain sickness (AMS) increases the levels of IL-1RA and HSP-70 when compared to placebo in patients susceptible to this disease [144]. In rats with adjuvant-induced arthritis, treatment with acetazolamide can inhibit secondary hindpaw swelling, alleviate ankle lesions, reduce the arthritis index, and lower the serum TNF-α and IL-1β levels [145,146] (Table 3).

### 2.4. Thiazide and Thiazide-like Diuretics

Thiazides inhibit the activity of the sodium–chloride co-transporter protein in the distal tubule of the nephron, which reduces the passage of sodium and chlorine from the lumen of the tubule into its epithelial cells. As a consequence, sodium ions and water that have accumulated in the body are eliminated. Moreover, the excretion of potassium and magnesium, as well as calcium retention, are intensified. Indications for the use of thiazides include heart failure, arterial hypertension, renal failure with a GFR > 30 mL/min/1.73 m^2^, and liver cirrhosis with ascites and edema. The contraindications to the use of thiazides are severe liver failure, severe electrolyte disturbances (hypokalemia, hyponatremia, and hypercalcemia), allergy to sulfonamides, and intoxication with cardiac glycosides. The most common side effects are electrolyte disturbances (hyponatraemia, hypokalemia, hypomagnesaemia, and hypercalcemia), dehydration and prerenal acute renal failure, a drop in blood pressure, an increased risk of thromboembolism, hyperglycemia, hypertriglyceridemia, an increase in LDL cholesterol, hyperuricemia, and gout, as well as gastrointestinal disturbances.

Thiazide diuretics reduce the infiltration of renal macrophages and slow the progression of renal disease [147]. However, hydrochlorothiazide does not affect TNF-α [148,149] or IL-1β production [150]. On the contrary, this drug inhibits the accumulation of T lymphocytes in tissues, especially in the thoracic lymph nodes, thoracic aorta, and kidney, in patients with hypertension [151,152]. Hydrochlorothiazide and chlorthalidone have been shown to decrease blood pressure, left ventricle hypertension, and proteinuria, but administration of these drugs does not affect reactive oxygen intermediate (ROI) or monocyte chemoattractant protein-1 (MCP-1) expression in blood vessels [153]. Similarly, bendroflumethiazide treatment does not show any effect on the TNF-α, IL-6, and TGF-β1 levels in mice [154]. Hydrochlorothiazide reduces IL-17A, which induces the remodeling of small arteries and increases blood pressure in mice [155].

Indapamide has properties that are very similar to hydrochlorothiazide and, like thiazide drugs, it acts in the final cortical segment of the ascending part of the Henle loop and in the initial part of the distal tubule. Its hypotensive effect is additionally related to the inhibition of calcium ion transport in smooth muscle cells, which results in their relaxation and vasodilation. Indapamide is used in the treatment of essential hypertension and edema caused by congestive heart failure. The pharmacological interaction and side effects are similar to those of other thiazides. Due to the fact that indapamide reduces the concentration of iodine bound to serum protein, it is contraindicated in patients with thyroid dysfunction. Indapamide slightly decreases the level of MCP-1 and macrophage inflammatory protein-1alpha (MIP-1alpha). Valsartan and indapamide have similar blood pressure-lowering effects, but valsartan has a more prominent effect on cytokine production [156]. Indapamide alleviates oxidative stress and inflammation in the renal cortex in rats by decreasing the expression of nuclear factor-κB and TGF-β1 [157] (Table 4).

### 2.5. The Most Recent Studies

The effect of diuretics and combined drugs (ACEI + diuretics) on the immune activity of murine macrophages has been investigated in CBA mice, showing that diuretics administered alone or with captopril change the proportion of cytokines in favor of anti-inflammatory cytokines (inhibitory effect on the production of pro-inflammatory cytokines (IL-6 and TNF-α), while the effect on anti-inflammatory cytokines (TGF-β1 and IL-10) is generally not statistically significant). Diuretics administered alone or with captopril increase the expression of surface markers that are important for the phagocytosis process (CD11b, CD16/CD32, and CD14) and the antigen presentation process (CD80, CD86, CD40, and MHC II). As for the macrophage-presenting activity, the generation of activated B cell SRBC (early humoral response) is increased by furosemide and hydrochlorothiazide treatment. Captopril does not affect the early response, but when added to furosemide it enhances it; however, when captopril is added to hydrochlorothiazide, it reduces the early humoral response. In the case of antibody formation, captopril (like furosemide and hydrochlorothiazide) enhances the maturation of antibodies through class switching. On the contrary, furosemide added to captopril enhances its effect, while hydrochlorothiazide added to captopril does not [158].

## 3. Conclusions and Future Perspectives

The substances that promote urination represent the most commonly used drugs in geriatric patients. Diuretics significantly impact the functions of immune cells and modulate the mechanisms of immune responses. The immunomodulatory effects of diuretics influence other inflammatory diseases that the patient has, e.g., metabolic and neuroendocrine diseases or depression. Diuretics should be used with antihypertensive drugs to enhance the beneficial systemic therapeutic hypotensive and immunomodulatory effect. It is also important to remember to reach a balance between the anti-inflammatory properties and protection against cancer and microbes in the therapy of inflammatory diseases. Based on the available literature, it is difficult to predict which of the studied diuretics show the strongest anti-inflammatory effect, because they have not been tested in one research model, but in many different research models by various researchers. The novelty of the article consists of a broad review of the latest literature, which covers both the experimental use of drugs in animal models and their clinical use in humans in very diverse disease and clinical circumstances.

In the absence of a single, comprehensive research model, a natural step on the path to an unequivocal answer regarding the influence of diuretics on individual subtypes of the immune response (humoral, cellular, and non-specific response) is to create a complex multi-center research project with a unified methodology to study this important research problem.

It is worth considering testing diuretics, as well as combined drugs (diuretics + hypotensive drugs). In the first phase, drugs should be tested in mice, followed by a human model. In an animal model, it is necessary to investigate the effect of the tested drugs on various elements of the immune response, such as the humoral and cellular responses and the non-specific immunological response. Clearly it is not possible to study the immune system effects of these drugs so extensively and unambiguously in humans, as geriatric patients usually take many other drugs that affect immune mechanisms. However, examining the essential inflammatory markers in humans should already provide information on the effect of these drugs in a human model. After combining the guidance obtained from both models (human and mice), we will obtain comprehensive and maximally objectified information on the impact of these drugs on the immune system.

## Figures and Tables

**Table 3:** The effect of carbonic anhydrase inhibitors on selected parts of the immune system. Abbreviations: HSP-70, 70 kilodalton heat shock proteins; IL-1RA, interleukin 1 receptor antagonist.

Drug	Immunological Mechanism (Reference)
Acethazolamide	Reduction in: - TNF-α production by mouse macrophages—so may thus may suppress inflammatory reactions [142,143]; - Serum TNF-α and IL-1β levels in rats with adjuvant-induced arthritis [145,146]. Increase in: - Levels of IL-1RA and HSP-70 [144].

**Table 4:** The effect of thiazide and thiazide-like diuretics on selected parts of the immune system. Abbreviations, ROI, reactive oxygen intermediate; MCP-1, monocyte chemoattractant protein-1; MIP-1alpha, macrophage inflammatory protein-1alpha; TGF-β1, transforming growth factor beta 1; IL, interleukin.

Drug Group	Immunological Mechanism (Reference)
Thiazide diuretics	Reduction in: - Infiltration of renal macrophages and the progression of renal disease [147].
Dichlorothiazide	Reduction in: - Dry weight of the inflammatory granuloma, which demonstrates anti-inflammatory properties—taken in long-term therapy with furosemide, dichlorothiazide, and ethacrynic acid in a rat model [79].
Hydrochlorotihiazide	Non-significant effect on: - Production of TNF-α [148,149] and IL-1β [150]; - ROI and MCP-1 expression [153]. Inhibition of: - Accumulation of T lymphocytes in patients with hypertension [151,152].
Chlorthalidone	Non-significant effect on: - ROI and MCP-1 expression [153].
Bendroflumethiazide	Reduction in: - Level of IL-17A [155]. Not significant effect on: - TNF-α, IL-6, and TGF-β1 levels in mice [154].
Indapamide	Reduction of: - Levels of MCP-1 and MIP-1alpha [156]; - Oxidative stress and inflammation in the renal cortex in rats by decreasing the expression of nuclear factor-κB and TGF-β1 [157].

## Data Availability

All data are included within the manuscript.

## References

[1] Muntner P., Carey R.M., Gidding S., Jones D.W., Taler S.J., Wright J.T., Whelton P.K. (2018). Potential US Population Impact of the 2017 ACC/AHA High Blood Pressure Guideline. Circulation.

[2] Yoon S.S., Gu Q., Nwankwo T., Wright J.D., Hong Y., Burt V. (2015). Trends in Blood Pressure among Adults with Hypertension: United States, 2003 to 2012. Hypertension.

[3] Whelton P.K., Carey R.M., Aronow W.S., Casey D.E., Collins K.J., Dennison Himmelfarb C., DePalma S.M., Gidding S., Jamerson K.A., Jones D.W. (2018). 2017 ACC/AHA/AAPA/ABC/ACPM/AGS/APhA/ASH/ASPC/NMA/PCNA Guideline for the Prevention, Detection, Evaluation, and Management of High Blood Pressure in Adults: A Report of the American College of Cardiology/American Heart Association Task Force on Clinical Practice Guidelines. Hypertension.

[4] Williams B., Mancia G., Spiering W., Agabiti Rosei E., Azizi M., Burnier M., Clement D.L., Coca A., de Simone G., Dominiczak A. (2018). ESC Scientific Document Group. 2018 ESC/ESH Guidelines for the Management of Arterial Hypertension. Eur. Heart J..

[5] Unger T., Borghi C., Charchar F., Khan N.A., Poulter N.R., Prabhakaran D., Ramirez A., Schlaich M., Stergiou G.S., Tomaszewski M. (2020). 2020 International Society of Hypertension Global Hypertension Practice Guidelines. J. Hypertens..

[6] Sonne-Holm S., Sørensen T.I., Jensen G., Schnohr P. (1989). Independent Effects of Weight Change and Attained Body Weight on Prevalence of Arterial Hypertension in Obese and Non-Obese Men. BMJ.

[7] Forman J.P., Stampfer M.J., Curhan G.C. (2009). Diet and Lifestyle Risk Factors Associated with Incident Hypertension in Women. JAMA.

[8] Wang N.-Y., Young J.H., Meoni L.A., Ford D.E., Erlinger T.P., Klag M.J. (2008). Blood Pressure Change and Risk of Hypertension Associated with Parental Hypertension: The Johns Hopkins Precursors Study. Arch. Intern. Med..

[9] Staessen J.A., Wang J., Bianchi G., Birkenhäger W.H. (2003). Essential Hypertension. Lancet.

[10] Ooi W.L., Budner N.S., Cohen H., Madhavan S., Alderman M.H. (1989). Impact of Race on Treatment Response and Cardiovascular Disease among Hypertensives. Hypertension.

[11] Otten M.W., Teutsch S.M., Williamson D.F., Marks J.S. (1990). The Effect of Known Risk Factors on the Excess Mortality of Black Adults in the United States. JAMA.

[12] Whelton P.K., Appel L.J., Espeland M.A., Applegate W.B., Ettinger W.H., Kostis J.B., Kumanyika S., Lacy C.R., Johnson K.C., Folmar S. (1998). Sodium Reduction and Weight Loss in the Treatment of Hypertension in Older Persons: A Randomized Controlled Trial of Nonpharmacologic Interventions in the Elderly (TONE). TONE Collaborative Research Group. JAMA.

[13] Graudal N.A., Hubeck-Graudal T., Jurgens G. (2017). Effects of Low Sodium Diet versus High Sodium Diet on Blood Pressure, Renin, Aldosterone, Catecholamines, Cholesterol, and Triglyceride. Cochrane Database Syst. Rev..

[14] Mozaffarian D., Fahimi S., Singh G.M., Micha R., Khatibzadeh S., Engell R.E., Lim S., Danaei G., Ezzati M., Powles J. (2014). Global Burden of Diseases Nutrition and Chronic Diseases Expert Group. Global Sodium Consumption and Death from Cardiovascular Causes. N. Engl. J. Med..

[15] Roerecke M., Kaczorowski J., Tobe S.W., Gmel G., Hasan O.S.M., Rehm J. (2017). The Effect of a Reduction in Alcohol Consumption on Blood Pressure: A Systematic Review and Meta-Analysis. Lancet Public Health.

[16] Carnethon M.R., Evans N.S., Church T.S., Lewis C.E., Schreiner P.J., Jacobs D.R., Sternfeld B., Sidney S. (2010). Joint Associations of Physical Activity and Aerobic Fitness on the Development of Incident Hypertension: Coronary Artery Risk Development in Young Adults. Hypertension.

[17] Woods J.W. (1988). Oral Contraceptives and Hypertension. Hypertension.

[18] Warner T.D., Mitchell J.A. (2008). COX-2 Selectivity Alone Does Not Define the Cardiovascular Risks Associated with Non-Steroidal Anti-Inflammatory Drugs. Lancet.

[19] Johnson A.G., Nguyen T.V., Day R.O. (1994). Do Nonsteroidal Anti-Inflammatory Drugs Affect Blood Pressure? A Meta-Analysis. Ann. Intern. Med..

[20] Pope J.E., Anderson J.J., Felson D.T. (1993). A Meta-Analysis of the Effects of Nonsteroidal Anti-Inflammatory Drugs on Blood Pressure. Arch. Intern. Med..

[21] Grover S.A., Coupal L., Zowall H. (2005). Treating Osteoarthritis with Cyclooxygenase-2-Specific Inhibitors: What Are the Benefits of Avoiding Blood Pressure Destabilization?. Hypertension.

[22] Bakris G.L., Ritz E. (2009). The Message for World Kidney Day 2009: Hypertension and Kidney Disease: A Marriage That Should Be Prevented. Kidney Int..

[23] Funder J.W., Carey R.M., Mantero F., Murad M.H., Reincke M., Shibata H., Stowasser M., Young W.F. (2016). The Management of Primary Aldosteronism: Case Detection, Diagnosis, and Treatment: An Endocrine Society Clinical Practice Guideline. J. Clin. Endocrinol. Metab..

[24] Young W.F. (2019). Diagnosis and Treatment of Primary Aldosteronism: Practical Clinical Perspectives. J. Intern. Med..

[25] Käyser S.C., Dekkers T., Groenewoud H.J., van der Wilt G.J., Carel Bakx J., van der Wel M.C., Hermus A.R., Lenders J.W., Deinum J. (2016). Study Heterogeneity and Estimation of Prevalence of Primary Aldosteronism: A Systematic Review and Meta-Regression Analysis. J. Clin. Endocrinol. Metab..

[26] Monticone S., Burrello J., Tizzani D., Bertello C., Viola A., Buffolo F., Gabetti L., Mengozzi G., Williams T.A., Rabbia F. (2017). Prevalence and Clinical Manifestations of Primary Aldosteronism Encountered in Primary Care Practice. J. Am. Coll. Cardiol..

[27] Dworkin L.D., Cooper C.J. (2009). Clinical Practice. Renal-Artery Stenosis. N. Engl. J. Med..

[28] Textor S.C., Lerman L. (2010). Renovascular Hypertension and Ischemic Nephropathy. Am. J. Hypertens..

[29] Peppard P.E., Young T., Palta M., Skatrud J. (2000). Prospective Study of the Association between Sleep-Disordered Breathing and Hypertension. N. Engl. J. Med..

[30] Punjabi N.M., Caffo B.S., Goodwin J.L., Gottlieb D.J., Newman A.B., O’Connor G.T., Rapoport D.M., Redline S., Resnick H.E., Robbins J.A. (2009). Sleep-Disordered Breathing and Mortality: A Prospective Cohort Study. PLoS Med..

[31] Gottlieb D.J., Yenokyan G., Newman A.B., O’Connor G.T., Punjabi N.M., Quan S.F., Redline S., Resnick H.E., Tong E.K., Diener-West M. (2010). Prospective Study of Obstructive Sleep Apnea and Incident Coronary Heart Disease and Heart Failure: The Sleep Heart Health Study. Circulation.

[32] Cadby G., McArdle N., Briffa T., Hillman D.R., Simpson L., Knuiman M., Hung J. (2015). Severity of OSA Is an Independent Predictor of Incident Atrial Fibrillation Hospitalization in a Large Sleep-Clinic Cohort. Chest.

[33] Redline S., Yenokyan G., Gottlieb D.J., Shahar E., O’Connor G.T., Resnick H.E., Diener-West M., Sanders M.H., Wolf P.A., Geraghty E.M. (2010). Obstructive Sleep Apnea-Hypopnea and Incident Stroke: The Sleep Heart Health Study. Am. J. Respir. Crit. Care Med..

[34] Young W.F. (2007). Adrenal Causes of Hypertension: Pheochromocytoma and Primary Aldosteronism. Rev. Endocr. Metab. Disord..

[35] Pacak K., Linehan W.M., Eisenhofer G., Walther M.M., Goldstein D.S. (2001). Recent Advances in Genetics, Diagnosis, Localization, and Treatment of Pheochromocytoma. Ann. Intern. Med..

[36] Stein P.P., Black H.R. (1991). A Simplified Diagnostic Approach to Pheochromocytoma. A Review of the Literature and Report of One Institution’s Experience. Medicine.

[37] Whitworth J.A. (1992). Adrenocorticotrophin and Steroid-Induced Hypertension in Humans. Kidney Int. Suppl..

[38] Saruta T., Suzuki H., Handa M., Igarashi Y., Kondo K., Senba S. (1986). Multiple Factors Contribute to the Pathogenesis of Hypertension in Cushing’s Syndrome. J. Clin. Endocrinol. Metab..

[39] Gumieniak O., Perlstein T.S., Hopkins P.N., Brown N.J., Murphey L.J., Jeunemaitre X., Hollenberg N.K., Williams G.H. (2004). Thyroid Function and Blood Pressure Homeostasis in Euthyroid Subjects. J. Clin. Endocrinol. Metab..

[40] Iglesias P., Acosta M., Sánchez R., Fernández-Reyes M.J., Mon C., Díez J.J. (2005). Ambulatory Blood Pressure Monitoring in Patients with Hyperthyroidism before and after Control of Thyroid Function. Clin. Endocrinol..

[41] Lind L., Hvarfner A., Palmér M., Grimelius L., Akerström G., Ljunghall S. (1991). Hypertension in Primary Hyperparathyroidism in Relation to Histopathology. Eur. J. Surg..

[42] Lind L., Ljunghall S. (1995). Pre-Operative Evaluation of Risk Factors for Complications in Patients with Primary Hyperparathyroidism. Eur. J. Clin. Investig..

[43] Yener Ozturk F., Erol S., Canat M.M., Karatas S., Kuzu I., Dogan Cakir S., Altuntas Y. (2016). Patients with Normocalcemic Primary Hyperparathyroidism May Have Similar Metabolic Profile as Hypercalcemic Patients. Endocr. J..

[44] Chen G., Xue Y., Zhang Q., Xue T., Yao J., Huang H., Liang J., Li L., Lin W., Lin L. (2015). Is Normocalcemic Primary Hyperparathyroidism Harmful or Harmless?. J. Clin. Endocrinol. Metab..

[45] (1997). The Sixth Report of the Joint National Committee on Prevention, Detection, Evaluation, and Treatment of High Blood Pressure. Arch. Intern. Med..

[46] Katz A.I. (1986). Distribution and Function of Classes of ATPases along the Nephron. Kidney Int..

[47] Wright F.S. (1982). Flow-Dependent Transport Processes: Filtration, Absorption, Secretion. Am. J. Physiol..

[48] Greger R., Velázquez H. (1987). The Cortical Thick Ascending Limb and Early Distal Convoluted Tubule in the Urinary Concentrating Mechanism. Kidney Int..

[49] Loon N.R., Wilcox C.S., Unwin R.J. (1989). Mechanism of Impaired Natriuretic Response to Furosemide during Prolonged Therapy. Kidney Int..

[50] Hropot M., Fowler N., Karlmark B., Giebisch G. (1985). Tubular Action of Diuretics: Distal Effects on Electrolyte Transport and Acidification. Kidney Int..

[51] Velázquez H., Wright F.S. (1986). Effects of Diuretic Drugs on Na, Cl, and K Transport by Rat Renal Distal Tubule. Am. J. Physiol..

[52] Kunau R.T., Weller D.R., Webb H.L. (1975). Clarification of the Site of Action of Chlorothiazide in the Rat Nephron. J. Clin. Investig..

[53] Plotkin M.D., Kaplan M.R., Verlander J.W., Lee W.S., Brown D., Poch E., Gullans S.R., Hebert S.C. (1996). Localization of the Thiazide Sensitive Na-Cl Cotransporter, RTSC1 in the Rat Kidney. Kidney Int..

[54] Ellison D.H., Velázquez H., Wright F.S. (1989). Adaptation of the Distal Convoluted Tubule of the Rat. Structural and Functional Effects of Dietary Salt Intake and Chronic Diuretic Infusion. J. Clin. Investig..

[55] Scherzer P., Wald H., Popovtzer M.M. (1987). Enhanced Glomerular Filtration and Na+-K+-ATPase with Furosemide Administration. Am. J. Physiol..

[56] Stanton B.A., Kaissling B. (1989). Regulation of Renal Ion Transport and Cell Growth by Sodium. Am. J. Physiol..

[57] Sheikhi A., Jaberi Y., Esmaeilzadeh A., Khani M., Moosaeefard M., Shafaqatian M. (2007). The Effect of Cardiovascular Drugs on Pro-Inflammatory Cytokine Secretion and Natural Killer Activity of Peripheral Blood Mononuclear Cells of Patients with Chronic Heart Failure in Vitro. Pak. J. Biol. Sci..

[58] Prandota J. (2002). Furosemide: Progress in Understanding Its Diuretic, Anti-Inflammatory, and Bronchodilating Mechanism of Action, and Use in the Treatment of Respiratory Tract Diseases. Am. J. Ther..

[59] Xu B., Makris A., Thornton C., Ogle R., Horvath J.S., Hennessy A. (2006). Antihypertensive Drugs Clonidine, Diazoxide, Hydralazine and Furosemide Regulate the Production of Cytokines by Placentas and Peripheral Blood Mononuclear Cells in Normal Pregnancy. J. Hypertens..

[60] Yuengsrigul A., Chin T.W., Nussbaum E. (1999). Immunosuppressive and Cytotoxic Effects of Furosemide on Human Peripheral Blood Mononuclear Cells. Ann. Allergy Asthma Immunol..

[61] Muniz-Junqueira M.I., Mota L.M., Aires R.B., Junqueira L.F. (2003). Digitalis Inhibits and Furosemide Does Not Change the in Vitro Phagocytic Function of Neutrophils of Healthy Subjects. Int. Immunopharmacol..

[62] Soloperto M., Marini M., Brasca C., Fasoli A., Mattoli S. (1991). The Protective Effect of Frusemide on the Generation of Superoxide Anions by Human Bronchial Epithelial Cells and Pulmonary Macrophages in Vitro. Pulm Pharmacol..

[63] Bianco S., Pieroni M.G., Refini R.M., Robuschi M., Vaghi A., Sestini P. (1993). Inhaled Loop Diuretics as Potential New Anti-Asthmatic Drugs. Eur. Respir. J..

[64] Wang S., Xiang Y.-Y., Ellis R., Wattie J., Feng M., Inman M.D., Lu W.-Y. (2011). Effects of Furosemide on Allergic Asthmatic Responses in Mice. Clin. Exp. Allergy.

[65] Murad H., Ghabrah T., Rafeeq M., Ali S. (2018). Subdiuretic Dose of Furosemide Enhances Albuterol Effects in Asthmatic Mice Rather than Bumetanide. Allergol. Immunopathol..

[66] Hofbauer R., Frass M., Pasching E., Gmeiner B., Kaye A.D., Kapiotis S. (2002). Furosemide and Spironolactone Reduce Transmigration of Leukocytes through Endothelial Cell Monolayers. J. Toxicol. Environ. Health A.

[67] Qu Q., Liu J., Zhou H.-H., Klaassen C.D. (2014). Nrf2 Protects against Furosemide-Induced Hepatotoxicity. Toxicology.

[68] Hirose K., Li S.-Z., Ohlemiller K.K., Ransohoff R.M. (2014). Systemic Lipopolysaccharide Induces Cochlear Inflammation and Exacerbates the Synergistic Ototoxicity of Kanamycin and Furosemide. J. Assoc. Res. Otolaryngol..

[69] Kroflic B., Coer A., Baudoin T., Kalogjera L. (2006). Topical Furosemide versus Oral Steroid in Preoperative Management of Nasal Polyposis. Eur. Arch. Otorhinolaryngol..

[70] Levasseur-Acker G.M., Molimard M., Regnard J., Naline E., Freche C., Lockhart A. (1994). Effect of Furosemide on Prostaglandin Synthesis by Human Nasal and Bronchial Epithelial Cells in Culture. Am. J. Respir. Cell Mol. Biol..

[71] Bernstein J.M. (2005). Update on the Molecular Biology of Nasal Polyposis. Otolaryngol. Clin. N. Am..

[72] Du Y., Li X., Liu B. (2014). Advances in Pathogenesis and Current Therapeutic Strategies for Cardiorenal Syndrome. Life Sci..

[73] Youssef M.I., Mahmoud A.A.A., Abdelghany R.H. (2015). A New Combination of Sitagliptin and Furosemide Protects against Remote Myocardial Injury Induced by Renal Ischemia/Reperfusion in Rats. Biochem. Pharmacol..

[74] Tuttolomondo A., Pinto A., Di Raimondo D., Corrao S., Di Sciacca R., Scaglione R., Caruso C., Licata G. (2011). Changes in Natriuretic Peptide and Cytokine Plasma Levels in Patients with Heart Failure, after Treatment with High Dose of Furosemide plus Hypertonic Saline Solution (HSS) and after a Saline Loading. Nutr. Metab. Cardiovasc. Dis..

[75] Tuttolomondo A., Di Raimondo D., Bellia C., Clemente G., Pecoraro R., Maida C., Simonetta I., Vassallo V., Di Bona D., Gulotta E. (2016). Immune-Inflammatory and Metabolic Effects of High Dose Furosemide plus Hypertonic Saline Solution (HSS) Treatment in Cirrhotic Subjects with Refractory Ascites. PLoS ONE.

[76] Tröger B., Heidemann M., Osthues I., Knaack D., Göpel W., Herting E., Knobloch J.K.-M., Härtel C. (2020). Modulation of S. Epidermidis-Induced Innate Immune Responses in Neonatal Whole Blood. J. Microbiol. Immunol. Infect..

[77] Rödler S., Roth M., Nauck M., Tamm M., Block L.H. (1995). Ca^2+^-Channel Blockers Modulate the Expression of Interleukin-6 and Interleukin-8 Genes in Human Vascular Smooth Muscle Cells. J. Mol. Cell. Cardiol..

[78] Nieminen L., Pyy K., Hirsimäki Y. (1976). The Effect of Furosemide on the Renal Damage Induced by Toxic Mushroom Cortinarius Speciosissimus in the Rat. Br. J. Exp. Pathol..

[79] Zverev I.F., Shelemba M.V., Belomestnykh V.G. (1985). Effect of diuretics on vascular permeability and proliferative inflammation in rats. Farmakol. Toksikol..

[80] Brennecke A., Villar L., Wang Z., Doyle L.M., Meek A., Reed M., Barden C., Weaver D.F. (2020). Is Inhaled Furosemide a Potential Therapeutic for COVID-19?. Am. J. Med. Sci..

[81] Veeraveedu P.T., Watanabe K., Ma M., Palaniyandi S.S., Yamaguchi K., Suzuki K., Kodama M., Aizawa Y. (2008). Torasemide, a Long-Acting Loop Diuretic, Reduces the Progression of Myocarditis to Dilated Cardiomyopathy. Eur. J. Pharmacol..

[82] Veeraveedu P.T., Watanabe K., Ma M., Thandavarayan R.A., Palaniyandi S.S., Yamaguchi K., Suzuki K., Kodama M., Aizawa Y. (2008). Comparative Effects of Torasemide and Furosemide in Rats with Heart Failure. Biochem. Pharmacol..

[83] Arumugam S., Sreedhar R., Miyashita S., Karuppagounder V., Thandavarayan R.A., Giridharan V.V., Pitchaimani V., Afrin R., Harima M., Suzuki K. (2014). Comparative Evaluation of Torasemide and Furosemide on Rats with Streptozotocin-Induced Diabetic Nephropathy. Exp. Mol. Pathol..

[84] Hung C.-M., Peng C.-K., Wu C.-P., Huang K.-L. (2018). Bumetanide Attenuates Acute Lung Injury by Suppressing Macrophage Activation. Biochem. Pharmacol..

[85] Wang M., Gorasiya S., Antoine D.J., Sitapara R.A., Wu W., Sharma L., Yang H., Ashby C.R., Vasudevan D., Zur M. (2015). The Compromise of Macrophage Functions by Hyperoxia Is Attenuated by Ethacrynic Acid via Inhibition of NF-ΚB-Mediated Release of High-Mobility Group Box-1. Am. J. Respir. Cell Mol. Biol..

[86] Han Y., Englert J.A., Delude R.L., Fink M.P. (2005). Ethacrynic Acid Inhibits Multiple Steps in the NF-KappaB Signaling Pathway. Shock.

[87] Harada T., Fink M., Cruz R.J. (2018). Ethacrynic Acid Decreases Expression of Proinflammatory Intestinal Wall Cytokines and Ameliorates Gastrointestinal Stasis in Murine Postoperative Ileus. Clinics.

[88] Hansen P.R., Rieneck K., Bendtzen K. (2004). Spironolactone Inhibits Production of Proinflammatory Cytokines by Human Mononuclear Cells. Immunol. Lett..

[89] Miura R., Nakamura K., Miura D., Miura A., Hisamatsu K., Kajiya M., Nagase S., Morita H., Fukushima Kusano K., Ohe T. (2006). Anti-Inflammatory Effect of Spironolactone on Human Peripheral Blood Mononuclear Cells. J. Pharmacol. Sci..

[90] Haas M.J., Jurado-Flores M., Hammoud R., Feng V., Gonzales K., Onstead-Haas L., Mooradian A.D. (2019). The Effects of Known Cardioprotective Drugs on Proinflammatory Cytokine Secretion from Human Coronary Artery Endothelial Cells. Am. J. Ther..

[91] Kato Y., Kamiya H., Koide N., Odkhuu E., Komatsu T., Dagvadorj J., Watarai A., Kondo M., Kato K., Nakamura J. (2014). Spironolactone Inhibits Production of Proinflammatory Mediators in Response to Lipopolysaccharide via Inactivation of Nuclear Factor-ΚB. Immunopharmacol. Immunotoxicol..

[92] Bendtzen K., Hansen P.R., Rieneck K., Spironolactone/Arthritis Study Group (2003). Spironolactone Inhibits Production of Proinflammatory Cytokines, Including Tumour Necrosis Factor-Alpha and Interferon-Gamma, and Has Potential in the Treatment of Arthritis. Clin. Exp. Immunol..

[93] Ji W.-J., Ma Y.-Q., Zhang X., Zhang L., Zhang Y.-D., Su C.-C., Xiang G.-A., Zhang M.-P., Lin Z.-C., Wei L.-Q. (2016). Inflammatory Monocyte/Macrophage Modulation by Liposome-Entrapped Spironolactone Ameliorates Acute Lung Injury in Mice. Nanomedicine.

[94] Sabbadin C., Calò L.A., Armanini D. (2016). The Story of Spironolactones from 1957 to Now: From Sodium Balance to Inflammation. G. Ital. Nefrol..

[95] Cuppone R., Del Vecchio S., Zanninelli G., Delle Monache M., Ulissi A., Tavanti A., Angeloni A., Ricci G.L. (1988). Lymphocyte Function Tests in Cirrhotic Patients under Treatment with Spironolactone and Potassium Canrenoate. J. Int. Med. Res..

[96] Besedovsky L., Born J., Lange T. (2012). Blockade of Mineralocorticoid Receptors Enhances Naïve T-Helper Cell Counts during Early Sleep in Humans. Brain Behav. Immun..

[97] Zhang L., Hao J.-B., Ren L.-S., Ding J.-L., Hao L.-R. (2014). The Aldosterone Receptor Antagonist Spironolactone Prevents Peritoneal Inflammation and Fibrosis. Lab. Investig..

[98] Martín-Fernández B., Rubio-Navarro A., Cortegano I., Ballesteros S., Alía M., Cannata-Ortiz P., Olivares-Álvaro E., Egido J., de Andrés B., Gaspar M.L. (2016). Aldosterone Induces Renal Fibrosis and Inflammatory M1-Macrophage Subtype via Mineralocorticoid Receptor in Rats. PLoS ONE.

[99] Mortensen L.A., Bistrup C., Stubbe J., Carlström M., Checa A., Wheelock C.E., Palarasah Y., Bladbjerg E.M., Thiesson H.C., Jensen B.L. (2019). Effect of Spironolactone for 1 Yr on Endothelial Function and Vascular Inflammation Biomarkers in Renal Transplant Recipients. Am. J. Physiol. Renal Physiol..

[100] Targoński R., Sadowski J., Price S., Targoński R. (2020). Sodium-Induced Inflammation-An Invisible Player in Resistant Hypertension. Hypertens. Res..

[101] Mareev V.Y., Orlova Y.A., Plisyk A.G., Pavlikova E.P., Matskeplishvili S.T., Akopyan Z.A., Seredenina E.M., Potapenko A.V., Agapov M.A., Asratyan D.A. (2020). Results of Open-Label Non-Randomized Comparative Clinical Trial: “BromhexIne and Spironolactone for CoronаvirUs Infection Requiring HospiTalization (BISCUIT). Kardiologiia.

[102] Esposito C.T., Varahan S., Jeyaraj D., Lu Y., Stambler B.S. (2013). Spironolactone Improves the Arrhythmogenic Substrate in Heart Failure by Preventing Ventricular Electrical Activation Delays Associated with Myocardial Interstitial Fibrosis and Inflammation. J. Cardiovasc. Electrophysiol..

[103] Elinoff J.M., Rame J.E., Forfia P.R., Hall M.K., Sun J., Gharib A.M., Abd-Elmoniem K., Graninger G., Harper B., Danner R.L. (2013). A Pilot Study of the Effect of Spironolactone Therapy on Exercise Capacity and Endothelial Dysfunction in Pulmonary Arterial Hypertension: Study Protocol for a Randomized Controlled Trial. Trials.

[104] Dinh Q.N., Young M.J., Evans M.A., Drummond G.R., Sobey C.G., Chrissobolis S. (2016). Aldosterone-Induced Oxidative Stress and Inflammation in the Brain Are Mediated by the Endothelial Cell Mineralocorticoid Receptor. Brain Res..

[105] Lieber G.B., Fernandez X., Mingo G.G., Jia Y., Caniga M., Gil M.A., Keshwani S., Woodhouse J.D., Cicmil M., Moy L.Y. (2013). Mineralocorticoid Receptor Antagonists Attenuate Pulmonary Inflammation and Bleomycin-Evoked Fibrosis in Rodent Models. Eur. J. Pharmacol..

[106] Nielsen S.E., Schjoedt K.J., Rossing K., Persson F., Schalkwijk C.G., Stehouwer C.D.A., Parving H.-H., Rossing P. (2013). Levels of NT-ProBNP, Markers of Low-Grade Inflammation, and Endothelial Dysfunction during Spironolactone Treatment in Patients with Diabetic Kidney Disease. J. Renin Angiotensin Aldosterone Syst..

[107] Biyashev D., Onay U.V., Dalal P., Demczuk M., Evans S., Techner J.-M., Lu K.Q. (2020). A Novel Treatment for Skin Repair Using a Combination of Spironolactone and Vitamin D3. Ann. N. Y. Acad. Sci..

[108] Lin M., Heizati M., Wang L., Nurula M., Yang Z., Wang Z., Abudoyreyimu R., Wu Z., Li N. (2021). A Systematic Review and Meta-Analysis of Effects of Spironolactone on Blood Pressure, Glucose, Lipids, Renal Function, Fibrosis and Inflammation in Patients with Hypertension and Diabetes. Blood Press..

[109] Myhre P.L., Vaduganathan M., O’Meara E., Claggett B.L., de Denus S., Jarolim P., Anand I.S., Pitt B., Rouleau J.L., Solomon S.D. (2020). Mechanistic Effects of Spironolactone on Cardiovascular and Renal Biomarkers in Heart Failure with Preserved Ejection Fraction: A TOPCAT Biorepository Study. Circ. Heart Fail..

[110] Barrera-Chimal J., Rocha L., Amador-Martínez I., Pérez-Villalva R., González R., Cortés-González C., Uribe N., Ramírez V., Berman N., Gamba G. (2019). Delayed Spironolactone Administration Prevents the Transition from Acute Kidney Injury to Chronic Kidney Disease through Improving Renal Inflammation. Nephrol. Dial. Transplant..

[111] Di Raimondo D., Tuttolomondo A., Buttà C., Miceli S., Licata G., Pinto A. (2012). Effects of ACE-Inhibitors and Angiotensin Receptor Blockers on Inflammation. Curr. Pharm. Des..

[112] Patel V., Joharapurkar A., Jain M. (2021). Role of Mineralocorticoid Receptor Antagonists in Kidney Diseases. Drug Dev. Res..

[113] Johnson L.A., Govani S.M., Joyce J.C., Waljee A.K., Gillespie B.W., Higgins P.D.R. (2012). Spironolactone and Colitis: Increased Mortality in Rodents and in Humans. Inflamm. Bowel Dis..

[114] McDonagh T.A., Metra M., Adamo M., Gardner R.S., Baumbach A., Böhm M., Burri H., Butler J., Čelutkienė J., Chioncel O. (2021). ESC Scientific Document Group. 2021 ESC Guidelines for the Diagnosis and Treatment of Acute and Chronic Heart Failure. Eur. Heart J..

[115] Fraccarollo D., Galuppo P., Schraut S., Kneitz S., van Rooijen N., Ertl G., Bauersachs J. (2008). Immediate Mineralocorticoid Receptor Blockade Improves Myocardial Infarct Healing by Modulation of the Inflammatory Response. Hypertension.

[116] Chen H., Sun F., Zhong X., Shao Y., Yoshimura A., Liu Y. (2013). Eplerenone-Mediated Aldosterone Blockade Prevents Renal Fibrosis by Reducing Renal Inflammation, Interstitial Cell Proliferation and Oxidative Stress. Kidney Blood Press. Res..

[117] Łabuzek K., Liber S., Bułdak Ł., Machnik G., Liber J., Okopień B. (2013). Eplerenone Promotes Alternative Activation in Human Monocyte-Derived Macrophages. Pharmacol. Rep..

[118] Xiong Y., Chang Y., Hao J., Zhang C., Yang F., Wang Z., Liu Y., Wang X., Mu S., Xu Q. (2021). Eplerenone Attenuates Fibrosis in the Contralateral Kidney of UUO Rats by Preventing Macrophage-to-Myofibroblast Transition. Front. Pharmacol..

[119] Du L., Qin M., Yi Y., Chen X., Jiang W., Zhou L., Zhang D., Xu K., Yang Y., Li C. (2017). Eplerenone Prevents Atrial Fibrosis via the TGF-β Signaling Pathway. Cardiology.

[120] Wada T., Ishikawa A., Watanabe E., Nakamura Y., Aruga Y., Hasegawa H., Onogi Y., Honda H., Nagai Y., Takatsu K. (2017). Eplerenone Prevented Obesity-Induced Inflammasome Activation and Glucose Intolerance. J. Endocrinol..

[121] Chi J.F., Uzui H., Guo H.Y., Ueda T., Lee J.D. (2014). Effects of Eplerenone on the Activation of Matrix Metalloproteinase-2 Stimulated by High Glucose and Interleukin-1β in Human Cardiac Fibroblasts. Genet. Mol. Res..

[122] Satoh M., Ishikawa Y., Minami Y., Akatsu T., Nakamura M. (2006). Eplerenone Inhibits Tumour Necrosis Factor Alpha Shedding Process by Tumour Necrosis Factor Alpha Converting Enzyme in Monocytes from Patients with Congestive Heart Failure. Heart.

[123] Zhou G., Johansson U., Peng X.-R., Bamberg K., Huang Y. (2016). An Additive Effect of Eplerenone to ACE Inhibitor on Slowing the Progression of Diabetic Nephropathy in the Db/Db Mice. Am. J. Transl. Res..

[124] Lax A., Sanchez-Mas J., Asensio-Lopez M.C., Fernandez-Del Palacio M.J., Caballero L., Garrido I.P., Pastor-Perez F.J., Januzzi J.L., Pascual-Figal D.A. (2015). Mineralocorticoid Receptor Antagonists Modulate Galectin-3 and Interleukin-33/ST2 Signaling in Left Ventricular Systolic Dysfunction after Acute Myocardial Infarction. JACC Heart Fail..

[125] Chen B., Geng J., Gao S.-X., Yue W.-W., Liu Q. (2018). Eplerenone Modulates Interleukin-33/SST2 Signaling and IL-1β in Left Ventricular Systolic Dysfunction After Acute Myocardial Infarction. J. Interferon Cytokine Res..

[126] Rafatian N., Westcott K.V., White R.A., Leenen F.H.H. (2014). Cardiac Macrophages and Apoptosis after Myocardial Infarction: Effects of Central MR Blockade. Am. J. Physiol. Regul. Integr. Comp. Physiol..

[127] Elinoff J.M., Chen L.-Y., Dougherty E.J., Awad K.S., Wang S., Biancotto A., Siddiqui A.H., Weir N.A., Cai R., Sun J. (2018). Spironolactone-Induced Degradation of the TFIIH Core Complex XPB Subunit Suppresses NF-ΚB and AP-1 Signalling. Cardiovasc. Res..

[128] Srinivasa S., Fitch K.V., Wong K., O’Malley T.K., Maehler P., Branch K.L., Looby S.E., Burdo T.H., Martinez-Salazar E.L., Torriani M. (2018). Randomized, Placebo-Controlled Trial to Evaluate Effects of Eplerenone on Metabolic and Inflammatory Indices in HIV. J. Clin. Endocrinol. Metab..

[129] Sun X.-N., Li C., Liu Y., Du L.-J., Zeng M.-R., Zheng X.-J., Zhang W.-C., Liu Y., Zhu M., Kong D. (2017). T-Cell Mineralocorticoid Receptor Controls Blood Pressure by Regulating Interferon-Gamma. Circ. Res..

[130] Terada Y., Ueda S., Hamada K., Shimamura Y., Ogata K., Inoue K., Taniguchi Y., Kagawa T., Horino T., Takao T. (2012). Aldosterone Stimulates Nuclear Factor-Kappa B Activity and Transcription of Intercellular Adhesion Molecule-1 and Connective Tissue Growth Factor in Rat Mesangial Cells via Serum- and Glucocorticoid-Inducible Protein Kinase-1. Clin. Exp. Nephrol..

[131] Tschöpe C., Van Linthout S., Jäger S., Arndt R., Trippel T., Müller I., Elsanhoury A., Rutschow S., Anker S.D., Schultheiss H.-P. (2020). Modulation of the Acute Defence Reaction by Eplerenone Prevents Cardiac Disease Progression in Viral Myocarditis. ESC Heart Fail..

[132] Zhang X., Liu J., Pang X., Zhao J., Wang S., Wu D. (2014). Aldosterone Induces C-Reactive Protein Expression via MR-ROS-MAPK-NF-ΚB Signal Pathway in Rat Vascular Smooth Muscle Cells. Mol. Cell. Endocrinol..

[133] Al-Kadi A., El-Daly M., El-Tahawy N.F.G., Khalifa M.M.A., Ahmed A.-S.F. (2021). Angiotensin Aldosterone Inhibitors Improve Survival and Ameliorate Kidney Injury Induced by Sepsis through Suppression of Inflammation and Apoptosis. Fundam. Clin. Pharmacol..

[134] Jover E., Matilla L., Garaikoetxea M., Fernández-Celis A., Muntendam P., Jaisser F., Rossignol P., López-Andrés N. (2021). Beneficial Effects of Mineralocorticoid Receptor Pathway Blockade against Endothelial Inflammation Induced by SARS-CoV-2 Spike Protein. Biomedicines.

[135] Maron B.A., Leopold J.A. (2014). The Role of the Renin-Angiotensin-Aldosterone System in the Pathobiology of Pulmonary Arterial Hypertension (2013 Grover Conference Series). Pulm. Circ..

[136] Xu K., Lin C., Ma D., Chen M., Zhou X., He Y., Moqbel S.A.A., Ma C., Wu L. (2021). Spironolactone Ameliorates Senescence and Calcification by Modulating Autophagy in Rat Tendon-Derived Stem Cells via the NF-ΚB/MAPK Pathway. Oxid. Med. Cell. Longev..

[137] Amador C.A., Barrientos V., Peña J., Herrada A.A., González M., Valdés S., Carrasco L., Alzamora R., Figueroa F., Kalergis A.M. (2014). Spironolactone Decreases DOCA-Salt-Induced Organ Damage by Blocking the Activation of T Helper 17 and the Downregulation of Regulatory T Lymphocytes. Hypertension.

[138] Németh Z.H., Mabley J.G., Deitch E.A., Szabó C., Haskó G. (2001). Inhibition of the Na^+^/H^+^ Antiporter Suppresses IL-12 P40 Production by Mouse Macrophages. Biochim. Biophys. Acta.

[139] Soliman M.M. (2011). Na^+^-H^+^ Exchange Blockade, Using Amiloride, Decreases the Inflammatory Response Following Hemorrhagic Shock and Resuscitation in Rats. Eur. J. Pharmacol..

[140] Mastronarde J.G., Monick M.M., Gross T.J., Hunninghake G.W. (1996). Amiloride Inhibits Cytokine Production in Epithelium Infected with Respiratory Syncytial Virus. Am. J. Physiol..

[141] Rolfe M.W., Kunkel S.L., Rowens B., Standiford T.J., Cragoe E.J., Strieter R.M. (1992). Suppression of Human Alveolar Macrophage-Derived Cytokines by Amiloride. Am. J. Respir. Cell Mol. Biol..

[142] West M.A., LeMieur T.L., Hackam D., Bellingham J., Claire L., Rodriguez J.L. (1998). Acetazolamide Treatment Prevents in Vitro Endotoxin-Stimulated Tumor Necrosis Factor Release in Mouse Macrophages. Shock.

[143] Hudalla H., Michael Z., Christodoulou N., Willis G.R., Fernandez-Gonzalez A., Filatava E.J., Dieffenbach P., Fredenburgh L.E., Stearman R.S., Geraci M.W. (2019). Carbonic Anhydrase Inhibition Ameliorates Inflammation and Experimental Pulmonary Hypertension. Am. J. Respir. Cell Mol. Biol..

[144] Julian C.G., Subudhi A.W., Wilson M.J., Dimmen A.C., Pecha T., Roach R.C. (2011). Acute Mountain Sickness, Inflammation, and Permeability: New Insights from a Blood Biomarker Study. J. Appl. Physiol..

[145] Cai L., Chen W.-N., Li R., Hu C.-M., Lei C., Li C.-M. (2018). Therapeutic Effect of Acetazolamide, an Aquaporin 1 Inhibitor, on Adjuvant-Induced Arthritis in Rats by Inhibiting NF-ΚB Signal Pathway. Immunopharmacol. Immunotoxicol..

[146] Cai L., Chen W.-N., Li R., Liu M.-M., Lei C., Li C.-M., Qiu Y.-Y. (2018). Acetazolamide Protects Rat Articular Chondrocytes from IL-1β-Induced Apoptosis by Inhibiting the Activation of NF-ΚB Signal Pathway. Can. J. Physiol. Pharmacol..

[147] Andrade-Oliveira V., Foresto-Neto O., Watanabe I.K.M., Zatz R., Câmara N.O.S. (2019). Inflammation in Renal Diseases: New and Old Players. Front. Pharmacol..

[148] Siragy H.M., Xue C., Webb R.L. (2006). Beneficial Effects of Combined Benazepril-Amlodipine on Cardiac Nitric Oxide, CGMP, and TNF-Alpha Production after Cardiac Ischemia. J. Cardiovasc. Pharmacol..

[149] Fukuzawa M., Satoh J., Ohta S., Takahashi K., Miyaguchi S., Qiang X., Sakata Y., Nakazawa T., Takizawa Y., Toyota T. (2000). Modulation of Tumor Necrosis Factor-Alpha Production with Anti-Hypertensive Drugs. Immunopharmacology.

[150] Nemati F., Rahbar-Roshandel N., Hosseini F., Mahmoudian M., Shafiei M. (2011). Anti-Inflammatory Effects of Anti-Hypertensive Agents: Influence on Interleukin-1β Secretion by Peripheral Blood Polymorphonuclear Leukocytes from Patients with Essential Hypertension. Clin. Exp. Hypertens..

[151] Itani H.A., McMaster W.G., Saleh M.A., Nazarewicz R.R., Mikolajczyk T.P., Kaszuba A.M., Konior A., Prejbisz A., Januszewicz A., Norlander A.E. (2016). Activation of Human T Cells in Hypertension: Studies of Humanized Mice and Hypertensive Humans. Hypertension.

[152] Marvar P.J., Thabet S.R., Guzik T.J., Lob H.E., McCann L.A., Weyand C., Gordon F.J., Harrison D.G. (2010). Central and Peripheral Mechanisms of T-Lymphocyte Activation and Vascular Inflammation Produced by Angiotensin II-Induced Hypertension. Circ. Res..

[153] Zhou M.-S., Schulman I.H., Jaimes E.A., Raij L. (2008). Thiazide Diuretics, Endothelial Function, and Vascular Oxidative Stress. J. Hypertens..

[154] Das S., Au E., Krazit S.T., Pandey K.N. (2010). Targeted Disruption of Guanylyl Cyclase-A/Natriuretic Peptide Receptor-A Gene Provokes Renal Fibrosis and Remodeling in Null Mutant Mice: Role of Proinflammatory Cytokines. Endocrinology.

[155] Orejudo M., García-Redondo A.B., Rodrigues-Diez R.R., Rodrigues-Díez R., Santos-Sanchez L., Tejera-Muñoz A., Egido J., Selgas R., Salaices M., Briones A.M. (2020). Interleukin-17A Induces Vascular Remodeling of Small Arteries and Blood Pressure Elevation. Clin. Sci..

[156] Xie Q., Wang Y., Sun Z., Yang T. (2006). Effects of Valsartan and Indapamide on Plasma Cytokines in Essential Hypertension. Zhong Nan Da Xue Xue Bao Yi Xue Ban.

[157] Ma F., Lin F., Chen C., Cheng J., Zeldin D.C., Wang Y., Wang D.W. (2013). Indapamide Lowers Blood Pressure by Increasing Production of Epoxyeicosatrienoic Acids in the Kidney. Mol. Pharmacol..

[158] Bryniarski P., Nazimek K., Marcinkiewicz J. (2021). Anti-Inflammatory Activities of Captopril and Diuretics on Macrophage Activity in Mouse Humoral Immune Response. Int. J. Mol. Sci..

